# Isotretinoin in Acne Vulgaris Complicated by Underlying Major Depression: A Case Report and Review of Literature

**DOI:** 10.1155/2021/9942327

**Published:** 2021-07-09

**Authors:** Matthew DeLuca, Maxsaya Baez Nuñez, Ezequiel Rodriguez, Krishan Chirimunj

**Affiliations:** ^1^St. George's University School of Medicine, True Blue, WI, Grenada; ^2^Department of Psychiatry, Bergen New Bridge Medical Center, 230 E Ridgewood Ave, Paramus, NJ 07652, USA

## Abstract

We relay the case of a teenage female with severe facial acne vulgaris and a past psychiatric history of major depressive disorder who presented to the emergency department with a primary complaint of ongoing suicidal ideation. Defining features of this case stem from the patient endorsing that her suicidal ideation was a result of her severe acne and the coinciding social perturbation it caused. Additionally, the patient reported that just four months prior to the current presentation, her dermatologist started her on isotretinoin therapy for the management of acne vulgaris. To the best of the authors' knowledge, there have been no reported cases which describe a teenage female presenting with active suicidal ideation secondary to severe acne vulgaris while concurrently undergoing treatment with isotretinoin. Given the controversial but reported association between isotretinoin and increased suicidality, we considered the appropriateness of continuing this medication for our patient. We then conducted a literature search evaluating the evidence concerning this association. In what follows, we present a unique case report and provide a thorough review of the evidence—or lack thereof—surrounding the relationship between isotretinoin and suicidality. Additionally, the authors aim to provide recommendations for the management of future patients who may present under similar circumstances.

## 1. Introduction

Isotretinoin (13-cis-retinoic acid) is a synthetic vitamin A analog that was approved for the treatment of severe acne by the US Food and Drug Administration (FDA) in 1982 [1, 2]. Since then, it has been regarded as an antiacne therapy that boasts excellent clinical efficacy and long-term results in even the most refractory cases. The therapeutic effects of isotretinoin derived from alterations to cell cycle progression and cellular differentiation [1, 2]. Despite its unparalleled success, the adverse effects of isotretinoin are among the most highly contested topics in psychiatric literature. Some adverse effects, such as teratogenicity and mucocutaneous xerosis, have been well documented. Others, however, such as the proposed relationship between isotretinoin and increased suicidal ideation, remain significantly debated to date. While a causal relationship between the two has not been established, the theory is often revived by case reports and case series on the topic. The mere contention of this adverse effect may lead to clinician hesitancy when considering the appropriateness of isotretinoin for use in vulnerable populations. As a result, the authors aim to explore the current literature and provide recommendations for patients such as ours, whose underlying core contributor of major depressive disorder and suicidal ideation were rooted in the debilitating effects of severe acne vulgaris.

## 2. Case Presentation

A teenage female with a past medical history of severe facial acne vulgaris and major depressive disorder with multiple suicide attempts and hospitalizations presented to the psychiatric emergency department after endorsing a suicidal statement to her psychologist. On examination, the patient displayed depressive symptoms of anhedonia, as well as feelings of hopelessness, helplessness, and worthlessness. Her mood was described as “sad,” and she endorsed passive suicidal ideation without a precise plan of execution. The patient reported that her suicidal ideation was largely a result of her severe acne vulgaris. Physical examination was remarkable for superficial, self-inflicted cuts that were present diffusely on her bilateral forearms. There was no reported family history of medical illness, psychiatric illness, or proposed genetic predisposition to disease.

Previous attempts to manage the patient's diagnosed major depressive disorder involved psychotropic trials of sertraline, escitalopram, fluoxetine, and duloxetine, which yielded negligible clinical improvement. Her first psychiatric hospitalization was two years prior to her current presentation, after she made a public display of her first attempted suicide. Medically, the patient was an otherwise healthy, well-developed female. Four months prior to her presentation to our facility, however, the patient's dermatologist started her on isotretinoin at a dose of 70 mg daily. Importantly, the patient did not feel that this medication was contributing to her sad mood or suicidal ideation. In fact, she endorsed the contrary, stating that her improved skin provided her with an increased level of confidence.

After an in-depth discussion with the patient and her family about treatment options, the patient and her parents voluntarily consented to a 48-hour psychiatric commitment to our inpatient pediatric unit. This allowed for the initiation of inpatient therapy and provided an opportunity for additional evaluation. The patient began cognitive behavioral therapy while inpatient and her medications were reviewed. The decision was made to continue the patient on isotretinoin in the setting of improving acne vulgaris with subsequent elevation in the patient's mood.

At the time of release, the patient was not found to be an immediate danger to herself, others, or property. Upon follow-up, the patient was psychiatrically stable and doing well. Of note, the patient's acne vulgaris was noted to be markedly improving throughout her follow-up appointments and isotretinoin was eventually discontinued by her dermatologist. Psychiatrically, the patient continued with her outpatient cognitive behavioral therapy, as well as with pharmacotherapy in the form of a single antidepressant for the management of her major depressive disorder.

## 3. Discussion

Our patient endorsed feelings of hopelessness, helplessness, and worthlessness, and suicidal ideation attributed to her severe acne vulgaris. We accounted for the fact that the patient's psychiatric symptoms significantly predated her isotretinoin use but did not precede her development of acne vulgaris. We also acknowledged the fact that she endorsed positive feelings towards the improvement it had on her appearance. The beneficial effects of isotretinoin typically occur after approximately four to six months of treatment [2]. The patient presented four months into her course of treatment, so consideration was given as to whether or not therapeutic efficacy had peaked. However, reflecting on the patient's improving mood prompted us to consider the possibility that addressing the underlying root contributor of the patient's distress—acne vulgaris—would significantly improve her major depression [3].

A subsequent literature search was conducted on available data pertaining to the association of isotretinoin and suicidality. Additionally, the literature evaluating an independent association between acne vulgaris and suicidality was also reviewed. Recent studies suggest that the occurrence of depression and suicide among individuals undergoing isotretinoin therapy has not been sufficiently demonstrated [4–17]. The limited available data suggests that the incidence of such cases may be no greater than that in the general population. Although the relationship has been suggested by case studies and therefore should not be immediately dismissed, it is rarely described. On the contrary, positive treatment results may have the potential to attenuate the psychiatric associations of acne vulgaris. As a result, the current literature suggests that for patients undergoing treatment with isotretinoin, withholding therapy because of the controversial association is not currently justified [6]. In fact, treatment has been shown to have likely attenuated suicidal behavior in addition to significantly improving quality of life [3, 18]. Furthermore, no established pharmacological mechanism could account for described presentation of psychiatric symptoms as a result of isotretinoin treatment [13].

A retrospective cohort study by Sundström et al. highlights the severity of the psychological burden of acne. Their results indicate that in the absence of effective resolution of acne there is an increased risk of suicide [18]. The study emphasizes that when prescribing isotretinoin, awareness of prior psychiatric symptomology should not be the sole determining factor when choosing a treatment modality. Notably, this was concluded while taking into account that suicide attempts may still be prevalent even after treatment has ended. For this reason, they suggest that close follow-up should continue for a minimum of one year following the completion of treatment. While treatment exposure could be an attributable factor, a more probable interpretation may be that the trigger for suicidal ideation stems from unmet expectations when acne resolution does not lead to concurrent improvement in the patient's social life [18].

A recent nationwide study by Droitcourt et al. suggested that there is a profound level of difficulty in correlating the continued rise in suicide risk with either the presence of acne vulgaris, or the proposed adverse effects of the isotretinoin [7]. We have, however, observed a noteworthy and sustained correlation between dermatological conditions and increased suicide risk. The available literature repeatedly indicates an increased suicide risk among populations whose dermatological conditions have affected their perception of body image, leading to clinically significant emotional distress [3, 19–29].

In conclusion, this thorough review of the current literature concerning the relationship between isotretinoin and suicidality has led us to suggest that, on balance, there is not sufficient evidence to justify acutely discontinuing isotretinoin in patients similar to ours, so long as a plan for consistent long-term monitoring and follow-up is in place [10, 14, 16]. Furthermore, we propose that when identified as a significant contributing factor to major depression and suicidal ideation, the treatment of severe acne vulgaris with isotretinoin should not be readily excluded as a result of the proposed association between its use and increased suicidality [10, 14, 16, 17, 19].

Limitations to this study include a relative paucity of data describing the unique treatment dilemma experienced during the care of our patient. Additionally, large-scale studies evaluating the independent relationship between acne vulgaris and suicide are similarly limited. It would be of value to conduct further research exploring the association between severe acne vulgaris and major depressive disorder with suicidal ideation. In summary, the available literature currently demonstrates a lack of sufficient evidence to justify the discontinuation of isotretinoin in future patients whose underlying root cause of major depressive disorder and suicidal ideation is identified as severe acne vulgaris.

## Data Availability

Readers can access the data supporting the conclusions of this study upon reasonable request.

## References

[1] Bremner J. D., Shearer K. D., McCaffery P. J. (2012). Retinoic acid and affective disorders: the evidence for an association. *The Journal of Clinical Psychiatry*.

[2] Layton A. (2009). The use of isotretinoin in acne. *Dermato-endocrinology*.

[3] Ortonne J. P. (1997). Oral isotretinoin treatment policy. Do we all agree?. *Dermatology*.

[4] Ludot M., Mouchabac S., Ferreri F. (2015). Inter-relationships between isotretinoin treatment and psychiatric disorders: depression, bipolar disorder, anxiety, psychosis and suicide risks. *World Journal of Psychiatry*.

[5] Nevoralová Z., Dvořáková D. (2013). Mood changes, depression and suicide risk during isotretinoin treatment: a prospective study. *International journal of dermatology*.

[6] Parker M., Dimity P., Wayne S. (2005). Isotretinoin, depression and suicide: a review of the evidence. *British Journal of General Practice*.

[7] Droitcourt C., Nowak E., Rault C. (2019). Risk of suicide attempt associated with isotretinoin: a nationwide cohort and nested case-time-control study. *International journal of epidemiology*.

[8] Celano C. M., Freudenreich O., Fernandez-Robles C., Stern T. A., Caro M. A., Huffman J. C. (2011). Depressogenic effects of medications: a review. *Dialogues in clinical neuroscience*.

[9] Marqueling A. L., Zane L. T. (2007). Depression and suicidal behavior in acne patients treated with isotretinoin: a systematic review. *Seminars in cutaneous medicine and surgery*.

[10] Samuels D. V., Rosenthal R., Lin R., Chaudhari S., Natsuaki M. N. (2020). Acne vulgaris and risk of depression and anxiety: a meta-analytic review. *Journal of the American Academy of Dermatology*.

[11] Rehn L. M. H., Meririnne E., Höök-Nikanne J., Isometsä E., Henriksson M. (2009). Depressive symptoms and suicidal ideation during isotretinoin treatment: a 12-week follow-up study of male Finnish military conscripts. *Journal of the European Academy of Dermatology and Venereology: JEADV*.

[12] McGrath E. J., Lovell C. R., Gillison F., Darvay A., Hickey J. R., Skevington S. M. (2010). A prospective trial of the effects of isotretinoin on quality of life and depressive symptoms. *The British journal of dermatology*.

[13] Hull P. R., D'Arcy C. (2003). Isotretinoin use and subsequent depression and suicide. *American journal of clinical dermatology*.

[14] Bozdağ K. E., Gülseren Ş., Güven F., Çam B. (2009). Evaluation of depressive symptoms in acne patients treated with isotretinoin. *The Journal of dermatological treatment*.

[15] Cohen J., Adams S., Patten S. (2007). No association found between patients receiving isotretinoin for acne and the development of depression in a Canadian prospective cohort. *The Canadian journal of clinical pharmacology = Journal canadien de pharmacologie clinique*.

[16] Hull P. R., D'Arcy C. (2005). Acne, depression, and suicide. *Dermatologic clinics*.

[17] Chia C. Y., Lane W., Chibnall J., Allen A., Siegfried E. (2005). Isotretinoin therapy and mood changes in adolescents with moderate to severe acne: a cohort study. *Archives of dermatology*.

[18] Sundström A., Alfredsson L., Sjölin-Forsberg G., Gerdén B., Bergman U., Jokinen J. (2010). Association of suicide attempts with acne and treatment with isotretinoin: retrospective Swedish cohort study. *BMJ*.

[19] Kaymak Y., Taner E., Taner Y. (2009). Comparison of depression, anxiety and life quality in acne vulgaris patients who were treated with either isotretinoin or topical agents. *International journal of dermatology*.

[20] Picardi A., Lega I., Tarolla E. (2013). Suicide risk in skin disorders. *Clinics in dermatology*.

[21] Barankin B., DeKoven J. (2002). Psychosocial effect of common skin diseases. *Canadian family physician Medecin de famille canadien*.

[22] Koo J. Y., Smith L. L. (1991). Psychologic aspects of acne. *Pediatric dermatology*.

[23] Fried R. G., Wechsler A. (2006). Psychological problems in the acne patient. *Dermatologic therapy*.

[24] Bowe W. P., Doyle A. K., Crerand C. E., Margolis D. J., Shalita A. R. (2011). Body image disturbance in patients with acne vulgaris. *The Journal of clinical and aesthetic dermatology*.

[25] Nguyen C. M., Koo J., Cordoro K. M. (2016). Psychodermatologic effects of atopic dermatitis and acne: a review on self-esteem and identity. *Pediatric dermatology*.

[26] Cotterill J. A., Cunliffe W. J. (1997). Suicide in dermatological patients. *The British journal of dermatology*.

[27] Uhlenhake E., Yentzer B. A., Feldman S. R. (2010). Acne vulgaris and depression: a retrospective examination. *Journal of cosmetic dermatology*.

[28] Hassan J., Grogan S., Clark-Carter D., Richards H., Yates V. M. (2009). The individual health burden of acne. *Journal of health psychology*.

[29] Halvorsen J. A., Stern R. S., Dalgard F., Thoresen M., Bjertness E., Lien L. (2011). Suicidal ideation, mental health problems, and social impairment are increased in adolescents with acne: a population-based study. *The Journal of investigative dermatology*.

