# Supplementary material for: Isotretinoin in Acne Vulgaris Complicated by Underlying Major Depression: A Case Report and Review of Literature
Source: Case Rep Psychiatry. 2021 Jul 9;2021:9942327. doi: 10.1155/2021/9942327 (PMC8286202; doi:10.1155/2021/9942327)
Supplement: Supplementary Materials — The CARE Checklist is provided. [file 9942327.f1.docx]

# **2013 CARE Checklist**

- **Title** – The diagnosis or intervention of primary focus followed by the words “case report”.
- **Key Words** – 2 to 5 key words that identify diagnoses or interventions in this case report (including "case report").
- **Abstract** – (structured or unstructured)
  - Introduction – What is unique about this case and what does it add to the scientific literature?
  - The patient’s main concerns and important clinical findings.
  - The primary diagnoses, interventions, and outcomes.
  - Conclusion – What are one or more “take-away” lessons from this case report?
- **Introduction** – Briefly summarizes why this case is unique and may include medical literature references.
- **Patient Information**
  - De-identified patient specific information.
  - Primary concerns and symptoms of the patient.
  - Medical, family, and psychosocial history including relevant genetic information.
  - Relevant past interventions and their outcomes.
- **Clinical Findings** – Describe significant physical examination (PE) and important clinical findings.
- **Timeline** – Historical and current information from this episode of care organized as a timeline (figure or table).
- **Diagnostic Assessment**
  - Diagnostic methods (PE, laboratory testing, imaging, surveys).
  - Diagnostic challenges.
  - Diagnosis (including other diagnoses considered).
  - Prognostic characteristics when applicable.
- **Therapeutic Intervention**
  - Types of therapeutic intervention (pharmacologic, surgical, preventive).
  - Administration of therapeutic intervention (dosage, strength, duration).
  - Changes in therapeutic interventions with explanations.
- **Follow-up and Outcomes**
  - Clinician- and patient-assessed outcomes if available.
  - Important follow-up diagnostic and other test results.
  - Intervention adherence and tolerability. (How was this assessed?)
  - Adverse and unanticipated events.
- **Discussion**
  - Strengths and limitations in your approach to this case.
  - Discussion of the relevant medical literature.
  - The rationale for your conclusions.
  - The primary “take-away” lessons from this case report (without references) in a one paragraph conclusion.
- **Patient Perspective** – The patient should share their perspective on the treatment(s) they received.
- **Informed Consent** – A multi-faceted approach was applied to the design of this case report in order to ensure the protection of patient confidentiality. All potentially identifying context, text, and imagery was omitted from both the case report and its corresponding discussion.
